# *Plasmodium falciparum* pre-erythrocytic stage vaccine development

**DOI:** 10.1186/s12936-020-3141-z

**Published:** 2020-02-03

**Authors:** Jessica Molina-Franky, Laura Cuy-Chaparro, Anny Camargo, César Reyes, Marcela Gómez, David Ricardo Salamanca, Manuel Alfonso Patarroyo, Manuel Elkin Patarroyo

**Affiliations:** 1 Health Sciences Faculty, Universidad de Boyacá, Tunja, Colombia; 2 Molecular Biology and Immunology Department, Fundación Instituto de Inmunología de Colombia (FIDIC), Bogotá, Colombia; 3 PhD Program in Biomedical and Biological Sciences, Universidad del Rosario, Bogotá, Colombia; 4 Animal Science Faculty, Universidad de Ciencias Aplicadas y Ambientales (U.D.C.A), Bogotá, Colombia; 5 3D Structures Department, Fundación Instituto de Inmunología de Colombia (FIDIC), Bogotá, Colombia; 6 Basic Sciences Department, School of Medicine and Health Sciences, Universidad del Rosario, Bogotá, Colombia; 7 Medical School, Universidad Nacional de Colombia, Bogotá, Colombia

**Keywords:** Malaria, Vaccine, Sporozoite, Clinical trial, Vaccine efficacy, Immune response

## Abstract

Worldwide strategies between 2010 and 2017 aimed at controlling malarial parasites (mainly *Plasmodium falciparum*) led to a reduction of just 18% regarding disease incidence rates. Many biologically-derived anti-malarial vaccine candidates have been developed to date; this has involved using many experimental animals, an immense amount of work and the investment of millions of dollars. This review provides an overview of the current state and the main results of clinical trials for sporozoite-targeting vaccines (i.e. the parasite stage infecting the liver) carried out by research groups in areas having variable malaria transmission rates. However, none has led to promising results regarding the effective control of the disease, thereby making it necessary to complement such efforts at finding/introducing new vaccine candidates by adopting a multi-epitope, multi-stage approach, based on minimal subunits of the main sporozoite proteins involved in the invasion of the liver.

## Background

Human malaria is a transmissible disease having high morbi-mortality worldwide; it is caused by five parasite species from the genus *Plasmodium*: *Plasmodium falciparum, Plasmodium vivax*, *Plasmodium ovale*, *Plasmodium malariae* and *Plasmodium knowlesi* (*P. falciparum* having the highest mortality rate) [1, 2].

Following the discovery of the parasite’s life-cycle which begins when the sporozoite (Spz) form is transmitted to humans during the bite of a female *Anopheles* mosquito [3], efforts at eliminating the disease became aimed at eliminating the vector and its habitats. After the failure of that strategy, the World Health Organization (WHO) efforts were aimed at promoting control programmes, which included long-lasting insecticide-treated mosquito nets and indoor spraying with residual insecticides, anti-malarial drug treatment and early and rapid diagnosis. Government entities in countries having malaria-endemic areas invest around 6.5 billion US dollars annually for controlling the disease worldwide [2].

Despite many efforts and scientific advances, the control and prevention of the disease has still not been achieved, as the WHO estimated 219 million cases of malaria and 435,000 malaria-related deaths for 2017, 93% of which were reported in sub-Saharan Africa, especially in children aged less than 5 years old and in pregnant women. It also estimated that the incidence rate between 2010 and 2017 had only become reduced by 18% [2]. Such statistics increasingly highlight the need for a global attack on malaria, including the development of an integral, multi-epitope, multi-stage, long-lasting vaccine able to induce a cellular and humoral immune response (IR) [4] as a fundamental, complementary and valuable tool for optimizing existing malaria control strategies. Contributing towards eliminating the disease would thereby help save hundreds of thousands of lives every year [2].

The female *Anopheles* mosquito injects a minimum of Spz (~ 100) during its bite [5, 6]; these remain at the inoculation site, moving in the dermis and seeking a capillary to enable them to migrate towards hepatocytes (having a high heparan sulphate proteoglycan (HSPG) content in their membrane) to invade them. This can last from 10 to 40 min, making Spz highly susceptible to a host IR, involving such a small amount of Spz and infected liver cells. This creates a bottleneck for the parasite during its reproductive cycle, making vaccines targeting Spz proteins and those from the parasite’s hepatic stage attractive vaccine candidates.

**Fig. 1:**
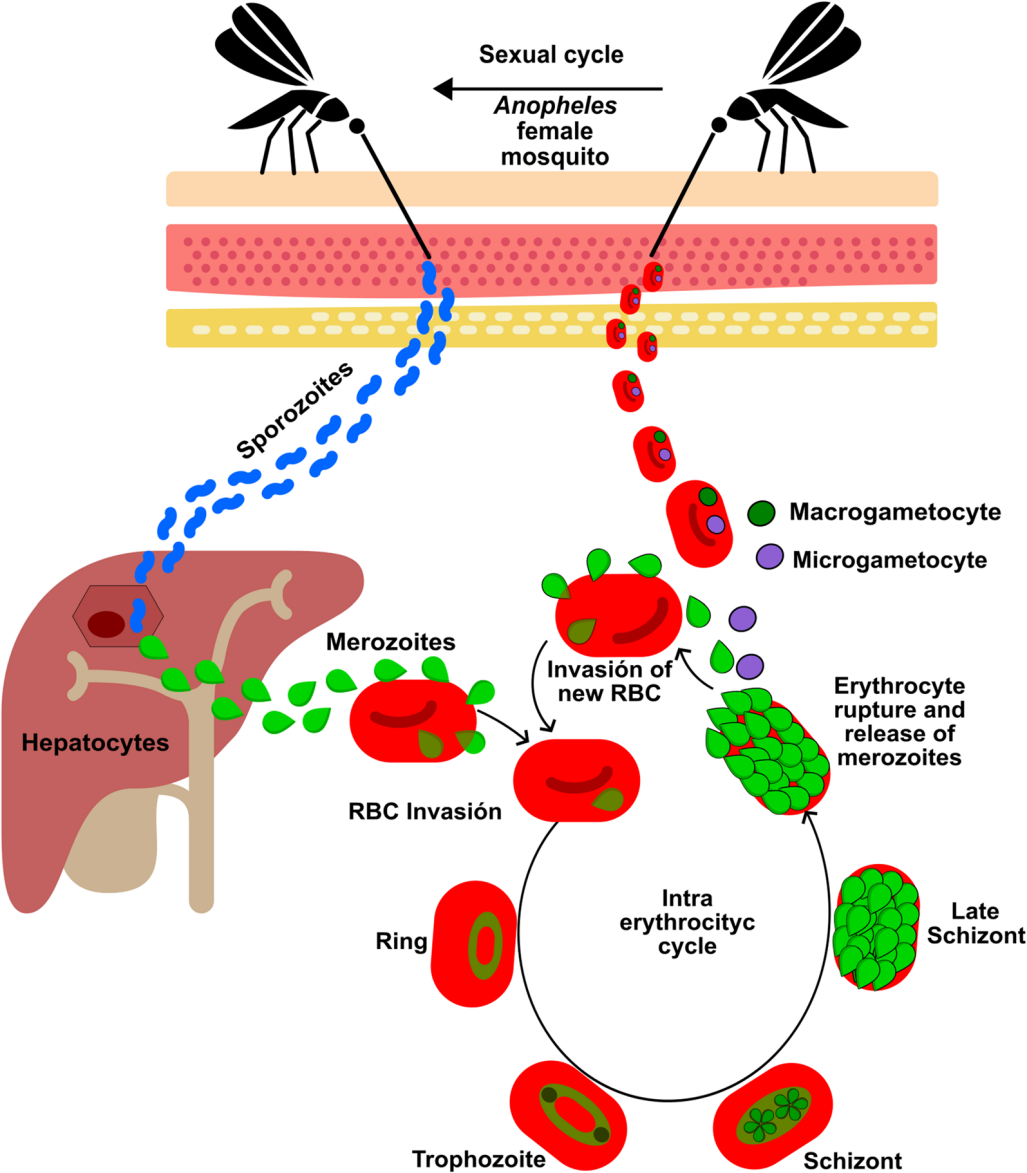
The *P. falciparum* life-cycle. An infected female *Anopheles* mosquito inoculates Spz as it bites a host, they then travel in the host’s bloodstream and infect the hepatocytes. Merozoites are released and then invade erythrocytes, where they mature through various stages (ring, trophozoite and schizont stages) and undergo asexual multiplication (~ 10 or lower) every 48 h, releasing new merozoites which perpetuate the asexual cycle. Some of them enter the sexual cycle by becoming female and male gametocytes which are ingested by the mosquito when it bites an infected host, thereby starting the cycle all over again

As this stage lasts 5.5 to 7 days, prolonging the length of exposure to the IR can detain infection, thereby hampering parasite development in the liver before symptoms appear during the blood stage, gametocyte production and the perpetuation of the parasite’s life-cycle (Fig. 1). Such fundamental approach complements vaccine candidates targeting the asexual erythrocyte stage during which millions of merozoites (Mrz) become exposed to the immune system during the extremely short period of time of around 1–2 min, thereby reducing the chances of success for such an approach [7, 8].

Based on prolonged IR exposure time, efforts have been focused on developing vaccines targeting Spz proteins. The WHO’s recent update [9] reported that vaccine candidates in clinical phase trials include attenuated Spz vaccines (radiation-attenuated Spz, Spz administered under drug coverage and genetically-attenuated Spz vaccines), recombinant protein vaccines (RTS,S and R21) and recombinant viral vectors vaccines (Chad63 MVA ME-TRAP, CSVAC, ChAd63 METRAP and MVA METRAP with the matrix-M adjuvant) (Table 1).

**Table 1:** Clinical phases for developing vaccines against sporozoite stage malaria

Participants	Dose	Efficacy	Refs.
Vaccines which involve using complete Spz			
Radiation-attenuated Spz PfSPZ (Phase I–II)			
80 adults (18–50 years-old)	Group I (n = 14): 4 doses of 7500 P*f*SPZ Group II (n = 22): 4 doses of 30,000 P*f*SPZ Group III (n = 22): 4 doses of 135,000 P*f*SPZ Group IV (n: 22): 4 or 6 doses of 135,000 P*f*SPZ	Group I and IV: 0% Group II: 2/16 participants	[6]
31 adults (18–45 years-old)	3 doses of 9.0 × 10^5^ P*f*SPZ IV every 8 weeks	Homologous challenge: 64% Heterologous challenge: 83%	[23]
108 adults (18 to 35 years-old)	Safety cohort: P*f*SPZ: 1.35 × 10^5^ on day 0 and 2.7 × 10^5^ on day 14 Main cohort: 2.7 × 10^5^ P*f*SPZ or SSN on days 0, 28, 56, 84 and 140	29%. Defining positive blood smears as having at least 2 *P. falciparum* parasites per 0.5 μl of blood	[24]
67 adults (18 to 35 years-old)	Group I (n:3): doses of 3 × 10^4^, 1.35 × 10^5^ and 2.7 × 10^5^ P*f*SPZ every 4 weeks Group II (n:23): 1.35 × 10^5^ P*f*SPZ Group III (n:24): 2.7 × 10^5^ P*f*SPZ (weeks 0, 4, 8, 12 and 20) Group IV (n:6): 2.7 × 10^5^ P*f*SPZ following the same scheme as for Group III Group V (n = 10): infectivity controls	Homologous CHMI: 4 out of 20 were protected (20%)	[25]
67 adults (18 to 45 years-old)	Group I and II (n:30): 2.7 × 10^5^ P*f*SPZ IV (weeks 0, 4, 8, 12 and 20) Group III (n:15): 4.5 × 10^5^ P*f*SPZ IV (weeks 0, 8 and 16) Infectivity control (n = 22)	Homologous CHMI Group I and II: 70% Group III: 57% Heterologous CHMI Group I and II: 10%	[27]
173 participants (6 months to 45 years-old)	Group I (n:18) Ia: 9 × 10^5^ P*f*SPZ Ib:1.8 × 10^6^ PfSPZ (weeks 0, 8 and 16) Group II (n:18) IIa: 9 × 10^5^ P*f*SPZ IIb:1.8 × 10^6^ P*f*SPZ (weeks 0, 8 and 16) Group III (n:18) IIIa: 9 × 10^5^ P*f*SPZ IIIb:1.8 × 10^6^ P*f*SPZ (weeks 0, 8 and 16) Group IV (n:18) IVa: 4.5 × 10^5^ P*f*SPZ IVb: 9.0 × 10^6^ P*f*SPZ (weeks 0, 8 and 16) Group V (n:21) Va: 2.7 × 10^5^ (week 0) P*f*SPZ Vb: 4.5 × 10^5^ P*f*SPZ Vc: 9 × 10^5^ P*f*SPZ	NE	[28]
Spz administered under drug coverage (Phase I–II)			
PfSPZ-CVac			
40 adults	Group I (n.9) 3 doses of 3.2 × 10^3^ PfSPZ Group II (n.9) 3 doses of 1.28 × 10^4^ PfSPZ Group III (n.9) 3 doses of 5.12 × 10^4^ PfSPZ Placebo (n.13)	Homologous CHMI Group I: 33% Group II: 67% Group III: 100%	[32]
CPS-CQ			
15 adults (18–45 years-old)	Group I (n.10) Group control (n.5) Spz administered under chloroquine coverage	Heterologous challenge Group I: 100%	[30]
CPS–CQ/CPS-MQ			
20 adults (19–35 years-old)	Group CPS-CQ (n.5) Group CPS-MQ (n.10) Group control (n.5)	Heterologous challenge 60%	[32]
Genetically-attenuated Spz vaccines (Phase I–II)			
6 adults (18–42 years-old)	Delivery of Pf p52−/p36− GAP SPZ via infected Anopheles mosquito bite	83%	[35]
10 adults	150 to 200 bites per subject Pf GAP3KO	100%	[36]
Recombinant protein vaccines			
RTS,S (Phase II)			
894 children (5–17 months-old)	3 doses of RTS,S/AS01E	56%	[67]
180 children (18 months-old to 4 years-old)	3 doses of RTS,S in 0.5 ml AS01E 3 doses of RTS,S in 0.5 ml AS01E	NE	[72]
511 infants (6–10 months)	3 doses of RTS,S/AS01E	59.1%	[73]
447 children (5–17 months-old)	3 doses of RTS,S/AS01	4.4%	[79]
RTS,S (Phase III)			
15,460 children (6 to 12 weeks-old and 5 to 17 months-old)	3 doses of RTS,S/AS01	Clinical malaria: 55.1%–Severe malaria: 34.8%	[77]
15,460 children (6 to 12 weeks-old and 5 to 17 months-old)	4 doses of RTS,S/AS01	(< 50,000 parasites/μl) 5–17 months-old: clinical malaria: 36.3%–severe malaria: 32.2% 6 to 12 weeks-old: clinical malaria: 25.9% Severe malaria: 17.3% 3 years: 0% 5 years-old: 48% and 56%	[78]
Recombinant viral vectors vaccines			
Chad63 MVA ME-TRAP (Phase I)			
54 adults	Group A (n:28): ChAd63 ME-TRAP increasing the dose from 1 × 10^8^ to 5 × 10^10^vp ID (groups 1-4) and from 1 × 10^10^ to 2 × 10^11^ IM (groups 5–7) Group B (n:26): ChAd63 ME-TRAP, followed at 8 weeks by MVA ME-TRAP and a booster dose for 5 volunteers with ChAd63 ME-TRAP and for 6 volunteers with MVA ME-TRAP	NE	[116]
36 adults (18–50 years-old)	Trial A (n:16): ChAd63 ME-TRAP n:6 1 × 10^10^ VP Trial B (n:30): 10^5^ × 10^10^ VP a the 56 days MVA ME-TRAP 2 × 10^8^ by intramuscular route (IM)	NE	[111]
138 children and infants	Gambia 2–6 year-olds Group 1a (n:6): 1 × 10^10^ vp ChAd63 ME-TRAP and 1 × 10^8^ pfu MVA ME-TRAP Group 1b (n:6): 1 × 10^10^ vp ChAd63 ME-TRAP and 2 × 10^8^ pfu MVA ME-TRAP Group 1c (n:6): 1 ml HDCRV Group 1d (n:6): 5 × 0^10^ vp ChAd63 ME-TRAP and 1 × 10^8^ pfu MVA ME-TRAP Group 1e (n:6): 5 × 10^10^ vp ChAd63 ME-TRAP and 2 × 10^8^ pfu MVA ME-TRAP Group 1f (n:6): 1 ml HDCRV Gambia 5–12 month-olds Group 2a (n:12): 1 × 10^10^ vp ChAd63 ME-TRAP and 1 × 10^8^ pfu MVA ME-TRAP Group 2b (n:12): 5 × 10^10^ vp ChAd63 ME-TRAP and 1 × 10^10^ pfu MVA ME-TRAP Group 2c (n:12): No vaccine Gambia 10 week-olds Group 3a (n:12): 1 × 10^10^ vp ChAd63 ME-TRAP and 1 × 10^8^ pfu MVA ME-TRAP Group 3b (n:12): 5 × 10^10^ vp ChAd63 ME-TRAP and 1 × 10^8^ pfu MVA ME-TRAP Group 3c (n:12): No vaccine Burkina Faso (n.30) 5–17 month-olds 5 × 10^10^ vp ChAd63 ME-TRAP and 1 × 10^8^ pfu MVA ME-TRAP	NE	[110]
Chad63 MVA ME-TRAP (Phase II)			
120 adults (18–50 years-old)	n. 120 ChAd63 ME-TRAP (5 × 10^5^ vp) after 8 weeks n. 60 cases: MVA ME-TRAP (2 × 10^8^ pfu) n. 60 controls: anti-rabies vaccine (0.5 ml)	8% adjusted efficacy: 50% (PCR positivity: more than 10 parasites per μl)	[117]
120 adults (18–50 years-old)	n. 120 ChAd63 ME-TRAP (5 × 10^5^ vp) after 8 weeks n. 60 cases: MVA ME-TRAP (2 × 10^8^ pfu) n. 60 controls: anti-rabies vaccine (0.5 ml)	67% (PCR positivity: more than 10 parasites per μl)	[118]
CSVAC (Phase I)			
24 adults (18–50 years-old)	Group 1a (n:4): 5 × 10^9^ vp ChAd63CS Group 1b (n:8): 5 × 10^9^ vp ChAd63CS–day 56 MVA CS 2 × 10^8^ pfu Group 2a (n:4): 5 × 10^10^ vp ChAd63CS Group 2b (n:8): 5 × 10^10^ vp ChAd63CS–day 56 MVA CS 2 × 10^8^ pfu	NE	[120]
36 adults (18–45 years-old)	Group 1 (n:15): ChAd63 CS 5 × 10^10^ vp–day 56 MVA CS 2 × 10^8^ UFP–Day 72 CHMI Group 2 (n:15): ChAd63 CS 5 × 10^10^ vp–day 56 MVA CS 2 × 10^8^ UFP–Day 72 CHMI Group 3 (n:6): Day 72 CHMI	NE	[121]
ChAd63/MVA ME-TRAP + Matrix M™ (Phase I)			
23 adults (18 to 50 years-old)	Control group (n:6): ChAd63 ME-TRAP 5 × 10^10^ vp, day 56 MVA ME-TRAP 2 × 10^8^ pfu Group II (n:9): ChAd63 ME-TRAP 5 × 10^10^ vp + Matrix-M 25 µg, day 56 MVA ME-TRAP 2 × 10^8^ pfu + Matrix-M 25 µg Group III (n:8): ChAd63 ME-TRAP 5 × 10^10^ vp + Matrix-M 50 µg, day 56 MVA ME-TRAP 2 × 10^8^ pfu + Matrix-M 50 µg	NE	[123]

*vp* viral particles, *pfu* plaque-forming units, *HDCRV* human diploid cell inactivated anti-rabies vaccine, *SSN* normal saline solution, *NE* not evaluated

This review has been aimed at analysing the formulation, dose, safety and immunogenicity of current clinical trials being carried out regarding vaccine candidates’ differing study phases, and including the structure of some protein fragments being studied.

## Clinical trials for pre-erythrocyte stage anti-malarial vaccines

The main thrust of research groups developing vaccines against the *P. falciparum* malaria Spz stage has involved Spz recombinant proteins, DNA or viral vectored protein fragments and attenuated Spz vaccines to induce malaria reactive CD4^+^ and CD8^+^ T-lymphocyte counts and high antibody (Abs) titres. Unfortunately, the most advanced candidate formulations to date have had limited efficacy. However, there have been significant developments regarding phase I, II and III trials (Table 1), which should prove useful for further vaccine development.

## Attenuated sporozoite vaccines

It has been demonstrated that Abs produced by immunization with whole, attenuated Spz prevent the development of hepatic infection and can immobilize free Spz in the avascular dermis or prevent erythrocyte stage development [10]. Vaccines based on this approach have included radiation-attenuated Spz (RAS), genetically-attenuated parasite (GAP) and Spz administered under drug coverage [11].

Many studies have been aimed at improving attenuated Spz vaccines, focusing on efforts at producing a large repertoire of immunogens, evaluating the impact of a particular regime, dosage and inoculation route, thereby enabling an effective cellular and humoral immune response to be achieved [12].

### Radiation-attenuated sporozoites

The *P. falciparum* Spz (*Pf*SPZ) vaccine is the main candidate containing live, radiation-attenuated, whole, aseptic and metabolically active Spz which have been isolated from the salivary glands of mosquitos infected by *P. falciparum* [13, 14]. Pioneering studies evaluated the effect of radiation on *Plasmodium berghei* Spz ability to invade and develop in mouse livers, demonstrating that infection became reduced with higher radiation doses [15] and that mice immunized with X-ray-irradiated *P*. *berghei* Spz became protected against homologous challenge and challenge with *Plasmodium vinckei* [16–19].

Clinical trials with attenuated Spz were carried out on 11 human volunteers based on the foregoing experimental findings; the volunteers were immunized with more than 1000 bites by irradiated mosquitos infected by Spz from the *P*. *falciparum* NF54 strain or 3D7/NF54 clone. All participants were protected against a first homologous challenge [20]; however, only 2/10 volunteers were protected against challenge with the *P. falciparum* 7G8 strain (heterologous challenge). Such results showed that attenuated Spz immunization could represent a good methodology for developing anti-malarial vaccine candidates, though involving the inconvenience of an impractical administration route despite having demonstrated 90% to 95% effectiveness concerning homologous challenge [20, 21].

It has been demonstrated that immunization by mosquito bite deposits Spz in the dermis and subcutaneous tissue; however, it has not yet been possible to replicate this by innoculation using a standard needle. This has led to many efforts at equalling the efficacy of the classical RAS vaccine, evaluating variables such as the delivery method, the inoculation route and the dose to be administered [10, 13, 22].

Recognizing this limitation, one study has evaluated the safety and immunogenicity of different doses of the *Pf*SPZ vaccine via subcutaneous (SC) vs. intradermal (ID) route. It reported that 2/16 volunteers in the group who had received 4 doses of 3 × 10^4^ *Pf*SPZ became protected and that protected volunteers, one immunized by ID and the other via SC, had T-cell responses to *Pf*SPZ and antibodies (200 and 800 titres) [13].

An open-label trial was performed to evaluate other administration routes in which 64% of volunteers became protected after homologous challenge with the *Pf* 3D7 strain clone in controlled human malaria infection (CHMI) 19 weeks (~ 4.5 months) later. Subjects who did not have parasitaemia were submitted to a repeat heterologous challenge 33 weeks (~ 8 months) after final immunization with the *P. falciparum* 7G8 heterologous strain, of these 83% remained without parasitaemia. These results suggested that the *Pf*SPZ vaccine could achieve limited but lasting protection against heterologous strains (~ 8 months or 33 weeks), although CD4^+^ and CD8^+^ T-cell responses did not increase, being limited after the second and third immunization [23].

The vaccine was well-tolerated in a clinical trial in Malí [24], having 29% efficacy against heterologous strains during 24-week (~ 6 month) follow-up without incurring in any serious local or systemic adverse events (AE). Effectiveness 3 to 24 weeks (~ 1 to 6 months) after the last immunization was evaluated by homologous intravenous CHMI which showed that 20% of the subjects who received 5 doses of 2.7 × 10^5^ *Pf*SPZ had become fully protected [25].

Promising results were obtained in homologous *Pf*SPZ CHMI prepared with NF54 strain Spz [26]. However, vaccine effectiveness became considerably reduced to 10% after challenge with the heterologous strain (no grade 3 or 4 AE being recorded) [27].

Immunization doses were increased to 9.0 × 10^5^ *Pf*SPZ and 1.8 × 10^6^ *Pf*SPZ in adolescents, children and infants aged 6 months old and older to assess the effects of the *Pf*SPZ dose and the immune response of children and infants who had been less exposed to *P. falciparum* compared to adults pre-exposed to long-term *P. falciparum* infection [28]. No significant differences were found in any age group regarding AE amongst vaccinated volunteers. On the other hand, it was found that most vaccinees developed antibodies (Abs) against *Pf*CSP when evaluating the humoral immune response, a higher response being observed in children aged 6 to 10 years old who had received 1.8 × 10^6^ *Pf*SPZ [28].

Higher Abs responses in children and infants who had been less exposed to *P. falciparum* [28] and subjects living in non-endemic areas [27] suggested that Africans’ reduced immune responses were due to immunoregulation following long-term exposure to *P. falciparum* infection [24, 25]. All such efforts have shown that *Pf*SPZ efficacy in adults who have not had prior exposure to *P. falciparum* depends on the administration route (to induce tissue resident T cells in the liver) and the dose (which determines the degree of protection durability against homologous and heterologous challenge). This highlights the need for an improved dosage strategy and/or an alternative vaccine approach in malaria-endemic areas [12].

It is expected that a phase III trial involving around 2100 people aged 2 to 50 years-old will begin in early 2020 on Bioko, an island off the Equatorial Guinea coast. The trial’s objective is to provide data regarding the necessary efficacy and safety for regulatory authorities’ approval. If the trial is successful, Sanaria intends to carry out another clinical trial involving a further 10,000 people on the island (Hoffman S, personal communication).

### Sporozoites administered under drug coverage

This approach has highlighted the fact that an anti-malarial vaccine based on immunization with live Spz and chemo-prophylactic cover of chloroquine (CPS-CQ) has achieved protection in 100% of the volunteers 8 weeks after the final immunization, such protection persisting for up to 2 years [29]. Furthermore, it has been reported that inducing high protection depends on the dose in homologous CHMI [29, 30].

Another trial which included live Spz evaluated chemo-prophylactic cover of mefloquine (CPS-MQ), finding similar safety and efficacy profiles (~ 60%) as those for CPS-CQ [31]. Moreover, IV administration of non-irradiated cryopreserved Spz to malaria-naive, healthy adult volunteers taking chloroquine as part of prophylactic anti-malarial treatment (vaccine approach denoted as PfSPZ-CVac) also gave 100% efficacy (9/9 volunteers) against homologous CHMI [32].

Different immunization regimens and pharmacological alternatives such as atovaquone/proguanil, azithromycin and pyrimethamine are currently being studied for developing safer and more effective methodological alternatives [22].

### Genetically-attenuated sporozoite vaccines

Another approach concerns genetic manipulation modifying, eliminating or attenuating genes from parasites and altering hepatic stage infection development [33]. Genetically attenuated parasite P36p gene-deficient Spz, have induced protection-inducing immunity against *P. berghei* in mice, demonstrating the lack of infection during blood stage [34].

The first clinical trial evaluating vaccine safety and immunogenicity in 6 volunteers who received p52 (−)/p36 (−) Spz GAP through the bites of infected *Anopheles* mosquitoes showed that the vaccine was well tolerated, having mild to moderate local and systemic reactions. Only 1 out of the 6 volunteers developed parasitaemia 12 days after exposure [35].

A phase I clinical trial, involving 10 volunteers tested the p52–/p36–/sap1– (*Pf*GAP3KO) vaccine lacking three genes expressed during *P. falciparum* pre-erythrocyte stage. This was administered by mosquito bite, mild to moderate AE being reported and the absence of parasitaemia up to day 28 after the last immunization. This demonstrated complete *Pf*GAP3KO attenuation, pre-erythrocyte development becoming arrested. Humoral immune response analysis showed that all subjects developed considerable IgG anti-circumsporozoite protein (CSP) titres [36], thereby supporting the claim that *Pf*GAP3KO is a safe and immunogenic candidate. Efficacy data is expected for this and another candidate involving genetically attenuated *P. falciparum* Spz (NF54 strain) (*Pf*SPZ-GA1) by eliminating the b9 gene and Spz and liver stage asparagine-rich protein. (SLARP) genes which are important for parasite development during liver stage [22, 37].

**Fig. 2:**
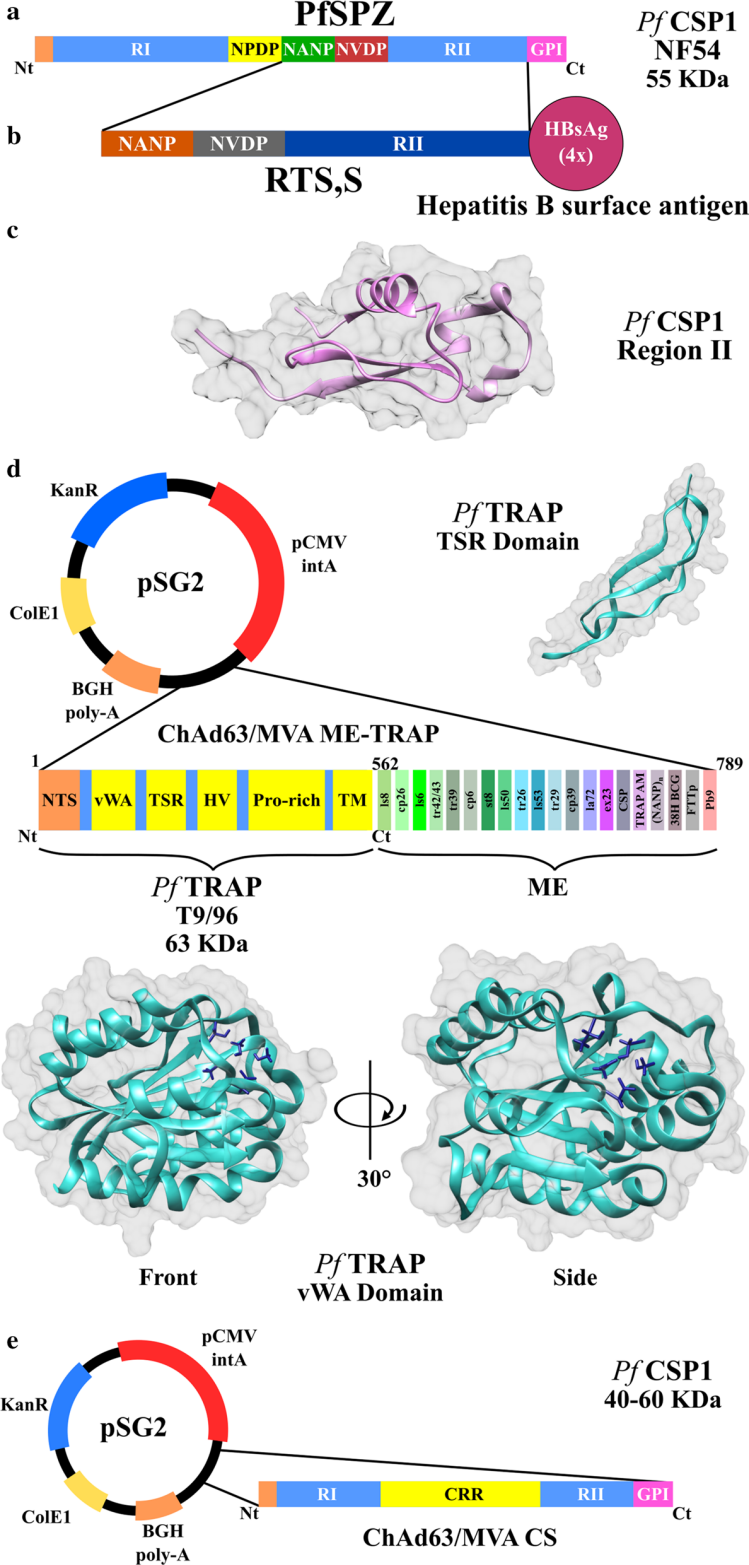
**a** Schematic representation of *P. falciparum’s* CSP1 (NF54 strain), showing signal peptide (orange), region I (blue), the central repeat regions (yellow, green and red) and region II (blue) with GPI anchor (pink). **b** Schematic representation of RTS,S vaccine, showing the central repeat regions (orange, grey) and PfCSP1 region II (blue) and hepatitis B virus (purple) surface antigen (S). **c** Ribbon and surface representation of PfCSP1 region II. (PDB: 3VDK) [177]. **d** Schematic representation of ChAd63/MVA ME-TRAP vaccine candidate. Left-hand side, above, pSG2 plasmid used to express the ME-TRAP vaccine candidate in either ChAd63 or MVA viruses involving kanamycine resistant (KanR) (in blue) cytomegalovirus, with intron A (pCMV IntA) (in red), bovine growth hormone with terminator polyA (BGH poly-A) (in orange) and *Escherichia coli* B-galactosidase genes (in yellow). Right-hand side, above, PfTRAP TRS domain in ribbon and surface representation (PDB 2BBX) [178]. Bottom, PfTRAP Von Willebrand factor A domain (vWA), in ribbon and surface, showing the MIDAS domain residues (blue). **e** pSG2 plasmid used to express the CS vaccine candidate in either ChAd63 or MVA viruses with the same vector as represented in E

*Plasmodium falciparum* CSP is located on Spz surface and is crucial for parasite morphogenesis and host invasion. It has variable length and 40 to 60 kDa molecular weight. It has an N-terminal domain containing region I, followed by a tandem repeat region consisting of the asparagine-alanine–asparagine-proline (NANP) amino acid (aa) motif repeated 20 to 40 times, interspaced four times with asparagine-valine-aspartate-proline (NVDP) and asparagine-proline-aspartate-proline (NPDP). It has a C-terminal domain (CTD), comprising region II and a glycosylphosphatidylinositol (GPI) anchor sequence [38, 39] (Fig. 2a, c).

An immunological response against NANP repeats has been a crucial point in developing CSP-basad vaccines. An analysis of the interaction between human monoclonal antibodies (mAbs) (RTS,S vaccine-derived 31, 317, Mal1C, Mal2A and Mal3B) and NANP repeats has led to identifying minimal epitope binding and confirming that an increase in the amount of Ab contacts can improve affinity for the repeats in this sequence [38, 40].

Recent studies have described mAbs CIS23, CIS34, CIS42 and CIS43 isolated from *P*. *falciparum* CSP-specific memory B-cells from volunteers who had been immunized with the *Pf*SPZ vaccine [41–43]. CIS43 and MGG4 mAb had cross-reactivity with NPDP, NVDP and NANP repeat regions and the CTD fragment, thereby enabling them to bind to this protein and alter its cleavage after processing to limit hepatocyte invasion in an animal model [42–44]. The next step will involve clinical trials being run by PATH’s Malaria Vaccine Initiative for determining whether mAbs can induce protection against *P*. *falciparum* infection.

## Recombinant protein vaccines

Recombinant vaccines can use one or multiple specific antigens to induce an immunological response against the parasite. They can be boosted when co-administered with adjuvants, thereby overcoming problems such as reverse virulence and the difficulty of obtaining sufficient amounts of the antigen to facilitate large-scale production [45]. However, using unsuitable antigens having low immunogenicity and a high genetic variation rate has limited the emergence of efficient vaccines against diseases such as malaria [46]. One of the main antigens involved in this approach has been *P. falciparum* CSP [11]  used as a subunit in the RTS,S vaccine.

### RTS,S

The RTS,S vaccine has been the most studied and publicized anti-malarial vaccine candidate in clinical phase trials according to WHO malaria vaccine guidelines [44]. RTS,S consists of a large segment (amino acids 207 to 395) of the *P. falciparum* NF54 strain CSP protein in which many variable epitopes has been identified [47, 48]. A tetrapeptide from the CSP NANP tandem repeat region (R) and the C-terminal region containing T-cell (T) epitopes (exclusive for the NF54 strain) become fused to hepatitis B surface (S) antigen (HBsAg) expressed in *Saccharomyces cerevisiae* yeast cells (Fig. 2b). These self-assemble into virus-like particles (VLP) and have a maximum 20% RTS sequence insertion into VLP [49].

The AS01 and AS02 adjuvant systems were well-tolerated and RTS,S/AS01 induced the highest anti-CSP and CD4^+^ T-cell responses, compared to RTS,S/AS02 when three doses were administered to children and infants instead of two doses [50–53]; these Abs persisted for at least three and a half years following immunization [54].

Anti-CSP antibody concentrations after a single RTS,S/AS02 booster dose (19 months after initial immunization), persisted for a further 5 years, even though titres became reduced to 4.7 μg/ml from levels preceding the booster dose [55]. Cellular and humoral immunological responses were associated, with protection-inducing responses against asymptomatic and symptomatic parasitaemia states [56, 57]. However, the considerable variation in such results was inexplicable; for example, children might have suffered malaria in spite of having had high anti-CSP titres [58].

Clinical trials have estimated that the vaccine had 30–86% efficacy following the last immunization using a standard three 50 μg dose scheme. However, this became reduced to 0% during the last weeks of follow-up [59–62].

Clinical evaluation results have suggested that RTS,S can be considered safe in spite of it inducing slight to moderate local reactogenicity, tending to escalate with an increase in dose regardless of age [49, 63, 64]. All doses were highly immunogenic, inducing anti-CSP and anti-HBsAg Abs, this being greater in children aged 1 to 5 years-old [65, 66]. Furthermore, it has been demonstrated that the inductor effect of RTS,S/AS02 protection is not associated with any particular Human Leukocyte Antigen (HLA) allele [60].

Safety and immunogenicity data have provided the basis for expanding the evaluation of new dosing strategies, vaccination schedules and extending the follow-up period, using larger samples of paediatric populations residing in malaria-endemic regions [49, 62].

Clinical trials in different aged paediatric populations have estimated 25.6–53% efficacy for at least 18 months’ follow-up and 0% after 3 years; this can be attributed to the intensity of transmission, the choice of adjuvant and the age of the population when being immunized [67–71]. However, significantly higher Abs responses have been reported after the third dose, even though these have not been long-lasting [72].

As the target population for immunization with RTS,S was infants, its safety and efficacy profile has been investigated due to being administered with other vaccines included in the Expanded Programme on Immunization (EPI) [73]. It was found that RTS,S did not interfere with the immunological responses of EPI antigens co-administered with it in infants [74] and that it had 52.5% efficacy against a first or single episode of malaria and 59.1% efficacy against all episodes during a 19-month period [73]. RTS,S/AS01E’s favourable safety profile suggested that the vaccine could be administered using a 0, 1 and 2 month scheme, which is why this scheme was chosen for a clinical evaluation in a multicentre phase III trial, delivering the vaccine via EPI. It was demonstrated that a scheme involving a complete dose of RTS,S at 0 and 1 months, together with a third fractioned dose at 7 months, increased protection against CHMI (86%) and improved immunogenicity by increasing specific antibody avidity and somatic hyper-mutation frequency in B-cells. The effect of changes in the vaccination scheme and the dose on protection-inducing immunity and vaccine efficacy must thus be studied in depth [75, 76].

A double blind, randomized controlled trial was carried out between 2009 and 2014 for evaluating RTS,S efficacy. It involved 15,460 participants divided into two age groups (6 to 12 week-olds and 5 to 17 month-olds) in 7 sub-Saharan Africa countries having different malaria transmission rates 14 months after the first vaccination, finding 34% efficacy against severe malaria in the combined age categories and 55.8% against clinical malaria in the 5 to 17 month-old group [77]. After 4 years follow-up, efficacy against episodes of clinical malaria was greater in the 5 to 17 month-old group (36.3%) compared to the 6 to 12 week-old group (25.9%) and against severe malaria (32.2% and 17.3%, respectively) [78].

It was found during a 7-year follow-up of a group of infants aged 5 to 17 months old who had received RTS,S that the efficacy of the vaccine against all episodes of malaria became reduced to − 3.6% in the fifth year and that average efficacy was 4.4% during the follow-up period [79]. Protection became reduced as time elapsed, becoming undetectable or exhibiting − 48% to − 56% negative efficacy during the last study period in the group which received three doses. This led to including a booster dose in the vaccination scheme after infants became 5 months old, considering that efficacy was lower in infants [78, 80].

RTS,S safety profile has been confirmed according to the data from phase I–III trials where local and systemic grade 3 AE incidence was low, study groups having similar frequency [78, 80, 81]. The fourth dose of RTS,S/AS01 was more reactogenic, having more systemic and local AE during the 7 days following vaccination compared to the group which received just three doses [58, 78]. Severe malaria incidence became reduced following vaccination with 50 µg RTS,S/AS01 in 3-year-old children in Tanzania, Kenya and Burkina Faso during 7-year follow-up, regardless of immunization scheme [82].

The European Medicines Agency (EMA) evaluated RTS,S’s clinical development in 2015, issuing a cautious scientific opinion regarding its quality [83], even though pre-clinical studies’ results are only being published 20 years after its clinical evaluation began. In a recently publicized trial, the WHO has recommended carrying out pilot introduction (with 360,000 participants) in three sub-Saharan countries (Kenya, Malawi and Ghana) having moderate to high levels of malarial transmission and only administering the four-dose scheme in the 5 to 17 month-old age group. It also suggested an initial scheme as being 3-dose, with a minimum 4-week interval between doses, followed by a 4th dose 15–18 months after the 3rd dose [84].

Several points regarding RTS,S have raised concern, such as high parasitaemia levels in individuals considered “protected” (> 5000 parasites/µl or 0.1% parasitaemia) [77, 78, 80] and the selected CSP region’s high genetic variability [85–88]. A not fully-defined adjuvant system has been used, mainly consisting of QS-21 (a saponin inducing cell activation through poorly understood mechanisms) [89–91], some RTS,S components have induced proapoptotic signals [92, 93] and it has had short-term efficacy [75, 78].

### R21

The R21 subunit-based vaccine is based on a single fusion protein; it consists of the *P. falciparum* NF54 strain CSP C-terminus bound to the HBsAg N-terminus. It has been developed as an improved version of RTS,S, containing a larger amount of CSP compared to HBsAg, promoting potent humoral immune responses to CSP and minimum Ab for the HBsAg portion. Efficacy against exposure to a transgenic Spz improved when BALB/c mice were given low doses of R21 [94].

A clinical trial carried out between 2015 and 2017 evaluated R21 safety and immunogenicity when administered with the ASO1 adjuvant; 20 healthy English participants received three doses of the vaccine on days 0, 28 and 56 of the trial. Good anti-CSP Ab responses were observed after a 6-month follow-up when using 10 μg and 50 μg doses, this being comparable with RTS,S levels induced against malaria. Both doses were well-tolerated, however there were safety-related AE. This study is registered in (ClinicalTrials.gov: NCT02600975), although no further information has been published.

## Recombinant viral vectors vaccines

Viral vectors represent promising tools for vaccine development, because they enable intracellular antigens to be expressed by increasing the ability to generate robust cytotoxic T-lymphocyte responses and proinflammatory interferon and cytokine production without the need for an adjuvant [95]. However, there is great concern regarding their genotoxicity due to possible viral genome integration; this has led to many efforts aimed at finding a high level of safety and efficacy.

Several viral [96–100], bacterial [101–104] and parasite [105–107] vectors have been used in anti-malarial vaccine candidates; currently, many clinical trials are exploring their advantages to increase their potential and accelerate their use in vaccines [11, 108].

### Chad63 MVA ME-TRAP

This anti-malarial vaccine was developed using chimpanzee adenovirus 63 (Chad63) and modified Vaccinia virus Ankara (MVA) into which were inserted genes encoding the thrombospondin-related adhesion protein (TRAP) multiple epitope (ME) chain [109, 110].

The ME-TRAP hybrid is thus a 2398 base pair (bp) insert encoding a single 789 aa-long peptide, covering the complete *P. falciparum* TRAP sequence, fused to a chain of 20 malaria T- and B-cell epitopes (14 targeting MHC class I, 3 MHC class II and 1 murine) (Fig. 2d) [111].

The MVA virus is highly attenuated and has been used efficiently as a non-replicating viral vector for developing new vaccines [112]. Chad63 serotypes do not circulate in human populations and thus neutralizing antibodies targeting them have seldom been demonstrated [113].

TRAP belongs to a family of proteins found in the micronemes during the invasion stages of parasites from the phylum Apicomplexa and in apical complex secretor vesicles. It is a 63 kDa, ~ 550 aa-long, conserved type I microneme protein, having two binding regions: the von Willebrand type A1 (VWA) region I, which includes the metal-ion-dependent-adhesion-site (MIDAS) and the TSR domain (region II), known for its role in protein–protein interactions. It also has a proline-rich region (region III), a transmembrane domain (region IV) and acidic C-terminal cytoplasmic tail (Fig. 2d) [114].

Sequential administration of MVA and Chad63 vectors, spaced by an interval of time (primary heterologous booster dose), is aimed at inducing CD4^+^ and CD8^+^ T-cells producing interferon gamma (IFN-ɣ) due to their main role in mediating protection during the hepatic stage [115].

A study with 54 participants, reported 184 local AE 28 days after initial vaccination (pain, erythema, oedema, pruritus and heat). All participants who received ID route vaccination reported local AE, lower incidence being reported by those who had received ChAd63 ME-TRAP by intramuscular (IM) route [116], thereby concluding that the ID route was associated with greater local reactogenicity compared to the IM route [111].

Systemic AE reported in a phase I study included fatigue (87%), general discomfort (69%) and fever (54%); 69% of them occurred and were resolved during the first 48 h after vaccination, increasing with vaccine dose regardless of administration route [116]. Such data is contrary to that described in another study where greater reactogenicity associated with vaccination route occurred (IM compared to ID) (i.e. no significant difference between doses) [110]. This study concluded that MVA ME-TRAP was more reactogenic than ChAd63 as it had greater AE incidence; however, both were well-tolerated [110].

Regarding the alterations reflected in the laboratory tests, there were increased transaminase levels following vaccination with ChAd63 ME-TRAP at the expense of alanine aminotransferase (ALT), eosinophilia and thrombocytopenia; this became resolved in 4 out of 54 participants [115]. This was contrary to that described in a study involving west-African children where no alterations in the participants’ haematological and biochemical tests were reported following vaccination [110].

A trial involving adults in Senegal [117] to assess vaccine efficacy using a polymerase chain reaction (PCR) assay was able to detect > 10 parasites/μl blood. PCR was positive for 12 out of 57 participants vaccinated with ChAd63 ME-TRAP with a booster dose of MVA ME-TRAP and 13 out of 58 control patients who received an anti-rabies vaccine were positive by PCR, giving 8% efficacy (which was not statistically significant). They thus grouped the results with the 67% efficacy obtained in a study in Kenya and, using Cox regression, showed 50% overall vaccine efficacy in both populations [117, 118].

### CSVAC

CSVAC, a vaccine from Chad63 and MVA to encode the *P. falciparum* CS protein, continued such line of research into plasmid DNA anti-malarial vaccines; the CS insert was a codon-optimized cDNA encoding the CS protein truncated at the C-terminal extreme thereby lacking 14 C-terminal aa and thus omitting the GPI anchor (Fig. 2f) [119].

No serious AE were found when evaluating this vaccine’s safety profile; 91% were slight and 80% were resolved within 48 h. It was found that 58% of the 24 volunteers had suffered one or more local AE following vaccination with ChAd63 CS compared to 83% of the volunteers suffering one or more systemic AE following vaccination, mostly affecting participants who had received 5 × 10^10^ vp ChAd56CS doses; it was concluded that MVA CS was more reactogenic in 87% of the volunteers [120].

The antigen-specific T-cell responses of two doses of ChAd63 CS were compared between group I (5 × 10^9^ vp) and group II (5 × 10^10^ vp) for evaluating immunogenicity. Reduced levels was reported up to day 56 (not statistically significant); responses in all volunteers increased significantly 7 days after administrating MVA CS, followed by a gradual decrease until follow-up day 140 [120].

CD4^+^ and CD8^+^ T-cell polyfunctionality was also evaluated, concluding that CD4^+^ produced greater TNF and IL2 levels, unlike IFNɣ values produced in similar amounts by CD4^+^ and CD8^+^ (no significant difference) [120].

All volunteers had IgG titres below the detection limit on day zero. The MVA CS booster dose produced a significant increase in Ab concentration on day 84 in group 1B compared to group 1A without booster dose; likewise, average Ab response was greater in group 2B compared to group 1B on day 140 (no statistically significant difference) (Table 1 gives detailed information about the groups) [120].

A CHMI study with *P. falciparum* Spz, involving a challenge which consisted of the infectious bites of 5 mosquitos evaluated vaccination efficacy by combining ChAd63/MVA CS with ChAd63/MVA ME-TRAP [121]. They reported that all infectivity controls (100%) and 27/30 (90%) of vaccinated participants were diagnosed with malaria and that 85% experienced at least one severe AE after challenge. They concluded that ME-TRAP had greater clinical efficacy by inducing sterile protection in 2 out of 15 participants (13%), unlike ChAd63/MVA CS which induced sterile protection in 1 out of 15 vaccinated participants (7%).

### ChAd63 METRAP and MVA METRAP with Matrix-M adjuvant

Vaccine candidates ChAd63 METRAP and MVA METRAP safety and immunogenicity have been evaluated when they have been administered with Matrix-M, a saponin-based adjuvant which stimulates the immune response and antigen presentation to local lymph nodes [122].

No increase in local reactogenicity was revealed in a phase I study involving 23 participants vaccinated with this adjuvant, pain in the inoculation area being the most commonly occurring local AE. More systemic AE were reported in the group which received the vaccine with the adjuvant, fever having greater prevalence in 8 volunteers (3 in the control group, 2 in the 25 µg Matrix-M group and 3 in the 50 µg Matrix-M group). Regarding cellular and humoral immunogenicity, there were no differences between control group and the group which received the vaccine with the adjuvant [123].

Considering the objective of using an adjuvant to boost an antigen-induced IR, the authors concluded that using the Matrix-M adjuvant had not lead to significant changes in vaccine immunogenicity [123].

## Future directions

Recent scientific advances have given rise to the need for safer formulations increasing antigen efficacy. “Nanovaccinology” has emerged during the last few years, which will surely come to play an important role in malaria vaccine development [124].

Using nanoparticles has enabled antigen stability, immunogenicity, selective administration and slow release to become improved [124]. Such characteristics have facilitated developing different vaccines from nanoparticles which have been approved for human use, varying in composition, form, surface properties and size (1–1000 nm) similar to cell components, enabling them to enter cells via mechanisms such as pinocytosis [125–127].

Nanoparticles have been used as delivery systems for vaccine candidates aimed at preventing disease caused by viral and bacterial, parasite and fungal pathogens [128–131], as well as non-infectious disease like cancer [132–134], Alzheimer’s [135], hypertension [136] and nicotine addiction [137]. Regarding parasitic diseases, CSP protein of *P. falciparum* has been encapsulated thereby enabling better Abs responses inhibiting the invasion of hepatocytes, inducing an immunological response which could contribute towards developing long-lasting protection-inducing immunity [138–141].

A promising alternative delivery system for subunit-based vaccines has been developed recently [134] and used with vaccine candidates against several infectious diseases such as HIV [142], toxoplasma [143–145], SARS [146], influenza [147] and/or malaria [148–150]. The technique is known as Self-Assembling Protein Nanoparticles (SAPNs) and involves the expression of a peptide/protein containing a target antigen covalently linked to an adjuvant sequence (flagellin-derived) and, in some cases, a universal epitope such as the Pan-DR T-helper epitope (PADRE) sequence. This peptide/protein can self-assemble in specific conditions, thus forming ~ 20–50 nm nanoparticles and, when formulated or emulsified with an adjuvant such as GLA-SE or Army Liposome Formulation (ALF), has managed to produce a protection-inducing response against several diseases [151, 152].

However, further studies are required to expedite understanding of how changes in nanoparticle properties might affect an immunological response against malaria and thus contribute towards effective vaccine design [153].

On the other hand, advances have been made in the fields of bioinformatics, genetic engineering and molecular biology, contributing towards using alternative methodological approaches. One such approach is reverse vaccinology for the relatively rapid identification of vaccine candidate molecules based on in silico analysis of complete sequences from the genomes of various pathogens for studying and evaluating their microbial biology and host–pathogen interactions [154–156]. Such methodology can be used with culturable and non-culturable microorganisms and, together with computational analysis, enables DNA sequences encoding proteins playing important roles in parasite biology to be identified and therefore become possible vaccine candidates [107, 108].

## Conclusions

The great scientific progress made regarding research into anti-malarial vaccine candidates over the last four decades has resulted from strategies promoted by scientific, academic and government institutions worldwide and extensive and generous support by official entities and philanthropic organizations clearly and deeply committed to resolving the malaria conundrum.

Current anti-malarial vaccine candidates have had limited efficacy due to the intrinsically complex problem and the multiple factors governing an appropriate immune response and the amount of external factors. The choice of antigen to be used is quite complicated due to factors such as the parasite’s complex life-cycle involving two reproduction cycles (sexual and asexual), different development stages and two hosts (the *Anopheles* mosquito and human beings). All this can be added to the multiple invasion routes described so far for each of its target cells (hepatocytes and/or erythrocytes), the parasite’s ability to modify its gene expression and the genetic variability between *P. falciparum* circulating strains [157–161].

Likewise, results to date have led to the conclusion that whole organism- or subunit-based vaccines involving a single parasite variant are insufficient to cover its wide genetic diversity.

Developing an anti-malarial vaccine based on sub-units derived from the proteins involved in parasite invasion and infection (multi-epitope) covering the parasite’s different forms (multistage) for overcoming such complications has been suggested for several decades now. Such subunits must consist of sequences which are conserved amongst *P. falciparum* circulating strains to induce a strain-transcending vaccine and overcome the parasite’s genetic variability [4, 114, 162, 163].

The next major challenge concerns the host’s genetic variability, particularly major histocompatibility class II (MHCII) complex molecules exerting their mechanism by synthesizing proteins encoded by the HLA-DR regions β1*, β3*, β4* and β5* where the HLA-DR β1* region encodes more than 1500 genetic variants grouped into 16 allele families called HLA-DRβ1*01, *03, *04, *07, etc. [164, 165]. Parasite proteins’ interaction with the human immune system should be analysed by predicting B and T epitopes (using NetMHCIIpan 3.2 or other predictors) and/or in vivo evaluation in models such as the *Aotus* monkeys (highly susceptible to developing human malaria and having a ~ 90% identical immune system with that of humans) [166–172].

Various adjuvants and delivery systems have been developed for improving vaccine efficacy. Clinical trials for Spz-stage anti-malarial vaccines have involved using adjuvants consisting of a combination of immunostimulants and viral vectors. The AS01 adjuvant has been used in RTS/S, consisting of a combination of immunostimulants, monophosphoryl lipid A (MPL) in a liposome formulation and *Quillaja saponaria* fraction 21 (QS21) in water-in-oil emulsion [91, 173].

Chimpanzee adenovirus (ChAd) has been developed as a vector due to concern at human adenoviruses’ pre-existing immunity and immunological potency [121, 174]. The vaccine involving a viral vector derived from serotype 63 ChAd (ChAd63) and modified vaccinia virus Ankara (MVA) has been widely evaluated in humans; it has been seen to be safe and a potent CD8^+^ T-cell and Ab inducer [116, 175, 176].

This review has thus described the great amount of knowledge accumulated to date whilst awaiting clinical phase results for the candidates described here, together with researchers’ other alternatives still being developed, as well as the difficulties and challenges still to be overcome as part of this long but fruitful way of developing vaccines. The target disease has been malaria, having a high global impact but, ideally, any approach demonstrating favourable results could be used regarding many other infectious diseases afflicting humanity.

## Data Availability

All data mentioned in this study are available in the referenced papers.

## Abbreviations

aa: Amino acid
Abs: Antibodies
AE: Adverse events
ALF: Army Liposome Formulation
ALT: Alanine aminotransferase
AS: Adjuvant system
Chad63: Chimpanzee adenovirus 63
CHMI: Controlled human malaria infection
CPS-CQ: Chemo-prophylactic cover of cloroquine
CPS-MQ: Chemo-prophylactic cover of mefloquine
CSP: Circumsporozoite protein
CTD: C-terminal domain
EMA: European Medicines Agency
EPI: Expanded Programme on Immunization
GAP: Genetically-attenuated parasite
GPI: Glycosylphosphatidylinositol
HBsAg: Hepatitis B surface antigen
HLA: Human leukocyte antigen
HSPG: High heparan sulphate proteoglycan
ID: Intradermal
IFN-ɣ: Interferon gamma
IgG: Immunoglobulin G
IR: Immune response
IV: Intravenous
mAbs: Monoclonal antibodies
ME: Multiple epitope
MHC: Major histocompatibility complex
MIDAS: Metal-ion-dependent-adhesion-site
MPL: Monophosphoryl lipid A
Mrz: Merozoites
MVA: Modified Vaccinia virus Ankara
NANP: Asparagine-alanine–asparagine-proline
NMRC: Naval Medical Research Center
NPDP: Asparagine-proline-aspartate-proline
NVDP: Asparagine-valine-aspartate-proline
PADRE: Pan-DR T-helper epitope
PCR: Polymerase chain reaction
PfSPZ: *P. falciparum* Spz
RAS: Radiation-attenuated Spz
SC: Subcutaneous
SFC: Spot-forming cell
SLARP: Liver stage asparagine-rich protein
Spz: Sporozoite
TRAP: Thrombospondin-related adhesion protein
VLP: Virus-like particle
vp: Viral particle
WHO: World Health Organization
